# Comparative Mitogenome Analysis Reveals Mitochondrial Genome Differentiation in Ectomycorrhizal and Asymbiotic *Amanita* Species

**DOI:** 10.3389/fmicb.2020.01382

**Published:** 2020-06-19

**Authors:** Qiang Li, Xiaohui He, Yuanhang Ren, Chuan Xiong, Xin Jin, Lianxin Peng, Wenli Huang

**Affiliations:** ^1^Key Laboratory of Coarse Cereal Processing, Ministry of Agriculture and Rural Affairs, College of Pharmacy and Biological Engineering, Chengdu University, Chengdu, China; ^2^Biotechnology and Nuclear Technology Research Institute, Sichuan Academy of Agricultural Sciences, Chengdu, China

**Keywords:** *Amanita*, mitochondrial genome, ectomycorrhiza, ecological adaptation, evolution

## Abstract

In this present study, we assembled and analyzed the mitogenomes of two asymbiotic and six ectomycorrhizal *Amanita* species based on next-generation sequencing data. The size of the eight *Amanita* mitogenomes ranged from 37,341 to 137,428 bp, and we considered introns to be one of the main factors contributing to the size variation of *Amanita*. The introns of the *cox1* gene experienced frequent gain/loss events in *Amanita*; and the intron position class cox1P386 was lost in the six ectomycorrhizal *Amanita* species. In addition, ectomycorrhizal *Amanita* species had more repetitive sequences and fewer intergenic sequences than asymbiotic *Amanita* species in their mitogenomes. Large-scale gene rearrangements were detected in the *Amanita* species we tested, including gene displacements and inversions. On the basis of the combined mitochondrial gene set, we reconstructed the phylogenetic relationships of 66 *Basidiomycetes*. The six ectomycorrhizal *Amanita* species were of single origin, and the two saprophytic *Amanita* species formed two distinct clades. This study is the first to elucidate the functions of the mitogenome in the evolution and ecological adaptation of *Amanita* species.

## Introduction

The genus of *Amanita*, belonging to *Agaricales*, *Basidiomycetes*, is a group of macrofungi. *Amanita* species are widely distributed around the world and have a variety of lifestyles (Wolfe et al., 2012a). Most of *Amanita* species form ectomycorrhizal associations with host plants, with some saprotrophic representatives (Wolfe et al., 2012b; De Mares et al., 2015). All *Amanita* species were derived from asymbiotic ancestors (De Mares et al., 2015). *Amanita* species has become one of the model organisms for the study of the life history, genetics, and evolution of ectomycorrhizal and saprotrophic fungi. Kohler et al. (2015) found that *Amanita* may lose some genes encoding plant cell wall–degrading enzymes in ectomycorrhizal life. Hess et al. (2014, 2018) further confirmed these results and proved that ectomycorrhizal symbiosis may be accompanied by an increased transposable element (TE) activity. Some ectomycorrhizal *Amanita* species have obtained carbohydrate metabolism genes through horizontal transfer (De Mares et al., 2015). The features and evolution of mitochondrial genomes (mitogenomes) in *Amanita* species remain unknown, however, which limits our comprehensive understanding of the evolution of *Amanita* species.

*Amanita* is a genus with a rich biodiversity, and approximately 600 species have been described in the genus (Sanchez-Ramirez et al., 2015). Consumers appreciate some species in this genus as delicious edible fungi, but some *Amanita* species are lethal, causing hundreds of cases of food poisoning every year worldwide (Cai et al., 2016; Ye and Liu, 2018). Similar and confusing morphological characteristics make it difficult to accurately distinguish between lethal and edible *Amanita* species based only on their morphologies (Garcia et al., 2015; Sun et al., 2018). The introduction of molecular markers, including internal transcribed spacers, RNA polymerase II, β-tubulin gene, and translation elongation factor 1-α gene (*tef1-*α), has promoted the classification and identification of *Amanita* species (Thongbai et al., 2016, 2017). The rapid rate of evolution, independent origin from the nuclear genome, and several available molecular markers has made the mitogenome a powerful tool for species classification and population genetics studies (Li et al., 2018b, c). Until now, however, no complete mitogenome has been published for the genus of *Amanita*.

Mitogenomes reportedly have been derived from alphabacteria through endosymbiosis by the ancestors of eukaryotes (Lang et al., 1999). By evolving with the host organisms, the features, gene arrangement, repeat sequences, introns, and protein-coding genes (PCGs) of mitogenomes have been considered to be useful information to reveal the evolution, environmental adaptation, and phylogeny of eukaryotic organisms (Boore, 1999; Poliseno et al., 2017; Qian et al., 2018; Sankoff et al., 1992). With the rapid development of the next-generation sequencing technology, increasing numbers of mitogenomes have been obtained, including animals, plants, and fungi. The mitogenome of fungi, however, has been less studied than that of animals, especially *Basidiomycetes*. Thus far, less than 100 *Basidiomycetes* mitogenomes were available in public databases [National Center for Biotechnology Information (NCBI)^1^ ], which has limited our comprehensive understanding of the genetics of the world’s largest mushroom-forming fungal group. Even relative to the total number of available nuclear genomes^2^, the number of *Basidiomycetes* mitogenomes was far behind. According to limited reports, the mitogenomes of *Basidiomycetes* were among the most variable mitogenomes. Their genome size, gene arrangement, content of repeat sequence, and intron classes varied greatly among different *Basidiomycetes* species as well as between closely related species (Kanzi et al., 2016; Sandor et al., 2018). The *Basidiomycetes* mitogenomes, however, were found to contain 14 conserved PCGs for energy metabolism (*atp6*, *atp8*, *atp9*, *cob*, *cox1*, *cox2*, *cox3*, *nad1*, *nad2*, *nad3*, *nad4*, *nad4L*, *nad5*, and *nad6*) and one *rps3* gene for transcriptional regulation, which we called the core PCGs of *Basidiomycetes* (Li et al., 2018d, 2019a). In addition, we also detected two rRNA genes and 20–36 tRNA genes in the *Basidiomycete* mitogenomes.

In the present study, we assembled and annotated mitogenomes of eight *Amanita* species, including six ectomycorrhizal species (*Amanita basii*, *Amanita muscaria*, *Amanita bisporigera*, *Amanita phalloides*, *Amanita brunnescens*, and *Amanita pseudoporphyria*) and two asymbiotic species (*Amanita inopinata* and *Amanita thiersii*). The aims of this study are (1) to reveal the features of *Amanita* mitogenomes and the similarities or variations between the ectomycorrhizal and asymbiotic mitogenomes in gene content, genome size, gene order, and repeat sequence; (2) to reveal the dynamic changes of introns in ectomycorrhizal and asymbiotic *Amanita* species. Mitogenomes of the eight *Amanita* species further our understanding of the evolutionary biology, genetics, and taxonomy of this important macrofungal genus.

## Materials and Methods

### Assembly and Annotations of Mitogenomes

We downloaded the raw sequencing data of *A. basii, A. muscaria, A. bisporigera, A. phalloides, A. brunnescens, A. inopinata*, and *A. thiersii* from the NCBI Sequence Read Archive (SRA) database (Hess et al., 2014, 2018; Kohler et al., 2015; Pulman et al., 2016). We obtained sequencing data for *A. pseudoporphyria* from our 100 mushroom genome project (Li H. et al., 2018). The species information and SRA accession numbers used for mitogenome assembly could be found in Supplementary Table S1. We conducted a series of quality control steps to obtain clean reads from the raw sequencing data. First, we removed adapter reads in the raw sequences using the AdapterRemoval v2 (Schubert et al., 2016), and then we filtered low-quality sequences using ngsShoRT (Chen C. et al., 2014). We used MITObim V1.9 (Hahn et al., 2013) to assemble the eight *Amanita* mitogenomes with the obtained clean reads. We annotated the obtained complete mitogenomes of the eight *Amanita* species according to our previously described methods (Li et al., 2018a). Briefly, we initially annotated the rRNA genes, tRNA genes, intron, and PCGs of the eight mitogenomes using the MITOS (Bernt et al., 2013) and MFannot (Valach et al., 2014), both of which are based on the genetic code 4. We then modified or predicted the PCGs using the NCBI Open Reading Frame Finder^3^ and further annotated the PCGs by BLASTP searches against the NCBI non-redundant protein sequence database (Bleasby and Wootton, 1990). We also used tRNAscan-SE v1.3.1 (Lowe and Chan, 2016) to identify or predict tRNA genes in the eight *Amanita* mitogenomes. We used OGDraw v1.2 (Lohse et al., 2013) to draw graphical maps of the eight complete mitogenomes.

### Sequence Analysis of the *Amanita* Mitogenomes

We used DNASTAR Lasergene v7.1^4^ to calculate base compositions of the eight *Amanita* mitogenomes. We assessed strand asymmetries of the eight mitogenomes according to the following formulas: AT skew = (A−T) /(A + T), and GC skew = (G−C)/(G + C) (Wang et al., 2017). Pairwise genetic distances between each pair of the 15 core PCGs (*atp6, atp8, atp9, cob, cox1, cox2, cox3, nad1, nad2, nad3, nad4, nad4L, nad5, nad6*, and *rps3*) were calculated using the MEGA v6.06 (Caspermeyer, 2016) with the Kimura-2-parameter (K2P) substitution model. Collinearity analysis of the eight *Amanita* mitogenome was conducted using Mauve (Darling et al., 2004), and color blocks of the same color represented homologous regions between different mitogenomes.

### Repetitive Element Analysis

To identify whether there were interspersed repeats or intragenomic duplications of large fragments throughout the eight mitogenomes, we conducted BLASTN searches of each mitogenome against itself using discontiguous megablast with general parameters (Johnson et al., 2008). We used Tandem Repeats Finder (Benson, 1999) with default parameters to detect tandem repeats (>10 bp in length) in the eight mitogenomes. We also used REPuter (Kurtz et al., 2001) to identify forward (direct), reverse, complemented, and palindromic (revere complemented) repeats in the eight mitogenomes. We manually calculated the content of non-overlapping repeat sequence identified by the three methods.

### Intron Analysis

We classified introns in *cox1* genes of the eight *Amanita* mitogenomes into different position classes (Pcls) according to the method described by Ferandon et al. (2010). Each Pcl was constituted by introns inserted at the same position in the coding region of the *cox1* gene. The same Pcl from different species usually had a high sequence similarity and contained orthologous intronic opening reading frames (ORFs). Pcls were named according to the method described by Zhang and Zhang (2019).

### Phylogenetic Analysis

To investigate the phylogenetic relationships of species in the Basidiomycota phylum, we constructed a phylogenetic tree of 67 species based on the combined mitochondrial gene set (15 core PCGs + 2 rRNA genes) (Li et al., 2018d). *Annulohypoxylon stygium* from the Ascomycota phylum was set as the outgroup (Deng et al., 2018). We used MAFFT v7.037 software (Katoh et al., 2017) to align individual mitochondrial genes and then used SequenceMatrix v1.7.8 (Vaidya et al., 2011) to concatenate these alignments into a combined mitochondrial gene set. We used the PartitionFinder 2.1.1 (Lanfear et al., 2017) to determine best-fit models of evolution and partitioning schemes for the gene set. We used Bayesian inference (BI) and maximum likelihood (ML) methods to construct phylogenetic trees. We conducted BI analysis using the MrBayes v3.2.6 (Ronquist et al., 2012) and performed ML analysis with RAxML v 8.0.0 (Stamatakis, 2014). When conducted BI analysis, two independent BI runs with four chains (three heated and one cold) were each conducted simultaneously over 2 × 10^6^ generations. Each run was sampled every 100 generations. Stationarity was assumed to have been reached when the estimated sample size (ESS) was greater than 100, and the potential scale reduction factor (PSRF) approached 1.0. The first 25% of the samples were discarded as burn-in, and the remaining trees were used to calculate Bayesian posterior probabilities (BPP) and construct a 50% majority-rule consensus tree.

### Data Availability

The complete mitogenomes of *A. basii, A. muscaria, A. bisporigera, A. phalloides, A. brunnescens, A. pseudoporphyria*, *A. inopinata*, and *A. thiersii* were deposited in the GenBank database under the accession numbers MK993555, MK993559, MK993556, MK993560, MK993557, MK993554, MK993558, and MK993561, respectively.

## Results

### Characterization and Protein-Coding Genes of *Amanita* Mitogenomes

**FIGURE 1:**
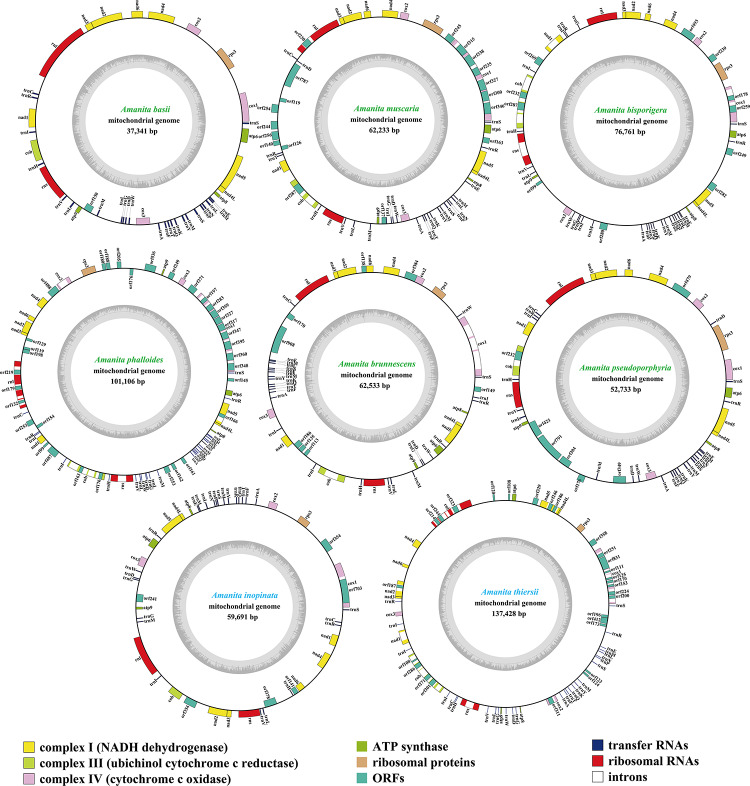
Circular maps of the mitogenomes of eight *Amanita* species. Genes are represented by different colored blocks. Colored blocks outside each ring indicate that the genes are on the direct strand, whereas colored blocks within the ring indicate that the genes are located on the reverse strand. The *Amanita* species with blue fonts are asymbiotic, while the species with green fonts are ectomycorrhizal.

The size of mitogenomes from eight *Amanita* species ranged from 37,341 to 137,428 bp, with an average size of 73,728 bp (Figure 1). The GC content of the eight mitogenomes ranged from 23.4 to 27.56%, and the average GC content was 24.63%. Of the eight mitogenomes we assembled, only three AT skews were positive, including *A. phalloides, A. inopinata*, and *A. thiersii*. The GC skews of all eight mitogenomes we tested were positive (Supplementary Table S1).

We detected 16–49 PCGs in the eight *Amanita* mitogenomes. *A. phalloides* contained the largest number of non-intronic PCGs among the eight *Amanita* species detected, with 34 (Supplementary Table S1); *A. basii* contained the least number of non-intronic PCGs, with only 16. All eight mitogenomes contained a set of core PCGs, including 14 core PCGs for energy metabolism and 1 *rps3* gene for transcriptional regulation (Supplementary Table S2). In addition, *A. phalloides*, *A. brunnescens*, *A. pseudoporphyria*, *A. inopinata*, and *A. thiersii* contained 2, 2, 3, 1, and 3 non-intronic genes coded DNA polymerases, respectively. *A. basii*, *A. muscaria*, *A. bisporigera*, *A. phalloides*, *A. brunnescens*, *A. pseudoporphyria*, *A. inopinata*, and *A. thiersii* contained 1, 10, 7, 17, 6, 3, 4, and 6 non-intronic genes with unknown functions, respectively. We detected 8, 4, 15, 1, 1, and 19 intronic ORFs detected in the mitogenomes of *A. muscaria*, *A. bisporigera*, *A. phalloides*, *A. pseudoporphyria*, *A. inopinata*, and *A. thiersii*, respectively. These intronic ORFs encoded homing endonucleases from different families, including the GIY-YIG homing nuclease and LAGLIDADG endonuclease.

### rRNA Genes and tRNA Genes

All eight *Amanita* mitogenomes contained two rRNA genes, namely, the large subunit ribosomal RNA (*rnl*) and the small subunit ribosomal RNA (*rns*) (Supplementary Table S2). The *rns* genes of *A. bisporigera*, *A. phalloides*, and *A. thiersii* all contained one intron, whereas the other *rns* genes did not contain any intron. The length of the *rns* gene varied in different *Amanita* species. *A. thiersii* had the longest *rns* gene, reaching 2,074 bp, whereas the *rns* gene of *A. inopinata* was the shortest, with only 1,896 bp. We detected 1, 4, and 4 introns in the *rnl* genes of *A. muscaria*, *A. phalloides*, and *A. thiersii*, respectively. *A. inopinata* had the longest *rnl* gene (3,552 bp), whereas *A. pseudoporphyria* had the shortest (3,367 bp).

We detected between 25 and 28 tRNA genes in the eight *Amanita* mitogenomes, which encoded 20 standard amino acids (Supplementary Table S2). All eight mitogenomes contained two tRNAs that coded for serine and leucine with different anticodons and three tRNAs that coded for methionine with the same anticodons. We also detected additional tRNA genes, including the trnD, trnG, trnI, and trnR, in the eight mitogenomes. The length of individual tRNAs ranged from 71 to 88 bp, with these length variations mainly resulting from the expansion or contraction of extra arms. The length expansions of extra arms in the *trns* genes contributed to their lengths becoming the longest among all the tRNAs.

### Repetitive Elements in *Amanita* Mitogenomes

By comparing the eight mitogenomes with themselves through a BLASTN search, we identified 5, 11, 64, 51, 56, 18, 8, and 34 repeat sequences in the mitogenomes of *A. basii, A. muscaria, A. bisporigera, A. phalloides, A. brunnescens, A. pseudoporphyria*, *A. inopinata*, and *A. thiersii*, respectively (Supplementary Table S3). The length of the individual repeat sequences ranged from 34 to 2,875 bp, with pair-wise nucleotide similarities ranging from 67.15 to 100%. The longest repeat sequences were located in the intergenic region between *orf99* and *cox3* and between the *orf280* and *trnA* genes in the *A. bisporigera* mitogenome. *A. bisporigera* contained the highest proportion of repetitive sequences, accounting for 36.62% of the entire mitogenome. Repeated sequences accounted for 2.53, 3.39, 10.26, 9.82, 12.36, 1.22, and 2.15% of the mitogenomes of *A. basii*, *A. muscaria*, *A. phalloides*, *A. brunnescens*, *A. pseudoporphyria*, *A. inopinata*, and *A. thiersii*, respectively.

We detected a total of 7, 15, 46, 56, 43, 7, 24, and 300 tandem repeats in the *A. basii, A. muscaria, A. bisporigera, A. phalloides, A. brunnescens, A. pseudoporphyria*, *A. inopinata*, and *A. thiersii* mitogenomes, respectively (Supplementary Table S4). The longest tandem sequence was located between *trnG* and *atp8* in the *A. thiersii* mitogenome, with a length of 258 bp. Most of the tandem repeat sequences were repeated two to four times in the eight *Amanita* mitogenomes, with the highest replication number (35) observed in the *A. thiersii* mitogenome. Tandem repeat sequences accounted for 1.31, 1.23, 3.31, 2.83, 3.53, 0.66, 1.66, and 14.83% of the *A. basii, A. muscaria, A. bisporigera, A. phalloides, A. brunnescens, A. pseudoporphyria*, *A. inopinata*, and *A. thiersii* mitogenomes, respectively. Using the REPuter, we identified 42 forward and eight reverse repeats in the mitogenome of *A. thiersii*, accounting for 3.44% of the entire mitogenome (Supplementary Table S5). Repeats identified by REPuter accounted for 2.92, 1.91, 3.84, 3.27, 4.50, 3.47, and 2.46 of the *A. basii, A. muscaria, A. bisporigera, A. phalloides, A. brunnescens, A. pseudoporphyria*, and *A. inopinata* mitogenomes, respectively.

### Phylogenetic Analysis

**FIGURE 2:**
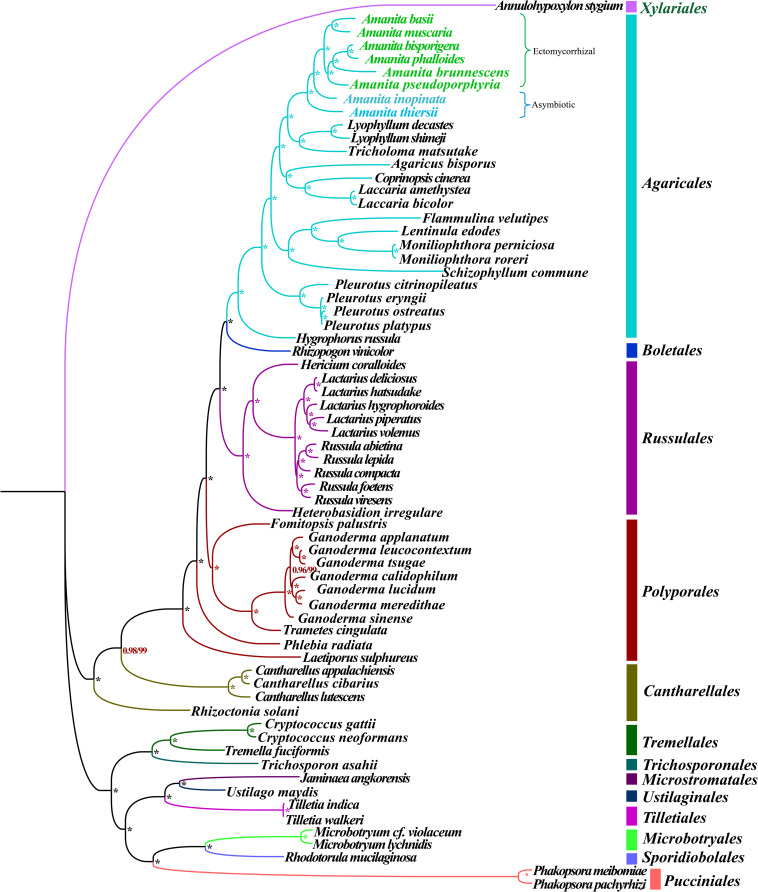
Molecular phylogeny of 66 *Basidiomycete* species based on BI and ML analysis of 15 PCGs and two rRNA genes. Support values are BPP (before slash) and BS values (after slash). The asterisk indicates that the BPP value is 1 and the BS value is 100 of the branch. Species and NCBI accession numbers for genomes used in the phylogenetic analysis are provided in Supplementary Table S6.

Phylogenetic analysis using ML and BI methods based on the combined mitochondrial gene set (14 core PCGs + *rps3* + 2 rRNA genes) using the GTR + I + G substitution model yielded identical and well-supported tree topologies (Figure 2). All major clades within the trees were well supported (BPP ≥0.96; bootstrap (BS) ≥99). On the basis of phylogenetic analysis, the 66 *Basidiomycota* species could be divided into 13 major clades, corresponding to the orders *Agaricales*, *Boletales*, *Russulales*, *Polyporales, Cantharellales*, *Tremellales*, *Trichosporonales*, *Microstromatales*, *Ustilaginales*, *Tilletiales*, *Microbotryales*, *Sporidiobolales*, and *Pucciniales* (Supplementary Table S6). The *Amanita* genus was divided into four groups, wherein the first included *A. thiersii*, the second group included *A. inopinata*, the third group was recovered as [*A. pseudoporphyria* + *A. brunnescens* + (*A. phalloides* + *A. bisporigera*)], and the forth group was recovered as (*A. muscaria* + *A. basii*). The phylogenetic analyses indicated that *A. phalloides* was a sister species to *A. bisporigera*, and *A. muscaria* was a sister species to *A. basii*. *A. thiersii* and *A. inopinata* were differentiated earlier from other *Amanita* species.

### Gene Rearrangement in *Amanita* Mitogenomes

**FIGURE 3:**
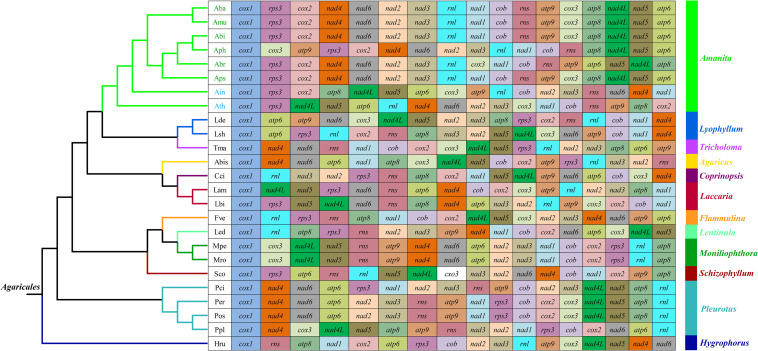
Mitochondrial gene arrangement analyses of 25 species from the genus of *Agaricales*. The phylogenetic positions of 25 *Agaricales* species were established using the BI method and ML method based on 15 concatenated mitochondrial core proteins and two rRNA genes. Genes are represented with different color blocks. All genes are shown in order of occurrence in the mitochondrial genome, starting from *cox1*. Fourteen core PCGs genes, one *rps3* gene, and two rRNA genes were included in the gene arrangement analysis. Species and NCBI accession number used for gene arrangement analysis in the present study are listed in Supplementary Table S6.

We frequently observed large-scale gene rearrangements at the level of families, even genera, from the *Agaricales* order (Li et al., 2018b, 2019b; Figure 3). The mitochondrial gene arrangements of the *Amanita* species were distinctly different from that of any other *Agaricales* species. Among the eight mitogenomes we tested, *A. basii, A. muscaria, A. bisporigera*, and *A. pseudoporphyria* had identical mitochondrial gene orders. Relative to the gene order in the four ectomycorrhizal *Amanita* species above, large-scale gene rearrangement events were found in the mitogenome of *A. phalloides, A. brunnescens, A. inopinata*, and *A. thiersii*. The displacement of *cox3* and *atp9* genes occurred in the mitogenome of *A. phalloides*. The mitogenome of *A. brunnescens* involved the displacement of *cox3* gene and the inversion of *atp6* and *nad5* with *nad4L* and *atp8* genes. Mitogenomes of *A. inopinata*, and *A. thiersii* involved multiple gene relocations, including *nad4L*, *nad5*, *atp6*, and *cox3*. The results indicated that the mitochondrial gene arrangement of *Amanita* species was diverse.

**FIGURE 4:**
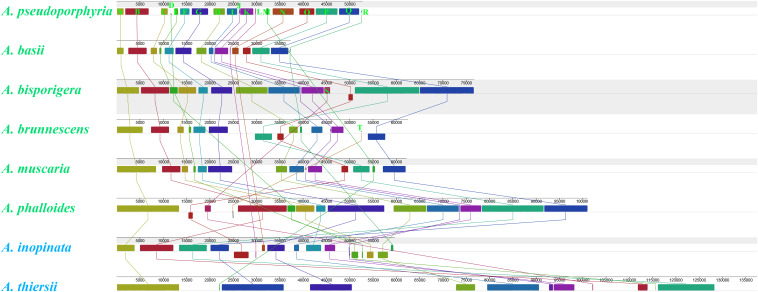
Colinearity analysis of eight *Amanita* mitogenomes using Mauve. Twenty homologous regions were detected among the eight mitogenomes. The sizes and relative positions of the homologous regions varied among mitogenomes. The *Amanita* species with blue fonts are asymbiotic, while the species with green fonts are ectomycorrhizal.

We detected 20 homologous regions among the eight *Amanita* mitogenomes (Figure 4). The size variation of homologous regions and the expansion or contraction of regions between homologous sequences promoted the size dynamics of *Amanita* mitogenomes. Different *Amanita* species contained varied amounts and groups of homologous regions. We detected homologous region N only in *A. basii*, *A. inopinata*, and *A. pseudoporphyria* mitogenomes; we detected homologous region S only in *A. bisporigera*, *A. phalloides*, and *A. thiersii*; and we detected homologous region T only in *A. brunnescens* and *A. phalloides* species. Mitogenomes of *A. basii*, *A. muscaria*, *A. bisporigera*, and *A. pseudoporphyria* exhibited a high degree of collinearity. We detected rearrangements of homologous regions in *A. phalloides, A. brunnescens, A. inopinata*, and *A. thiersii* species, of which the two asymbiotic species (i.e., *A. inopinata*, and *A. thiersii*) had experienced large-scale rearrangements of homologous regions.

### Variation and Genetic Distance of Core Genes

**FIGURE 5:**
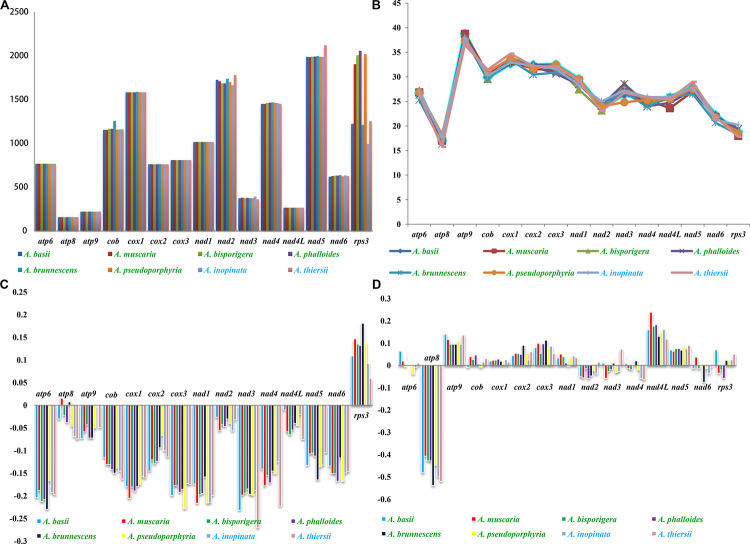
Variation in the length and base composition of each of the 15 PCGs among the eight *Amanita* mitogenomes. The *Amanita* species with blue fonts are asymbiotic, while the species with green fonts are ectomycorrhizal. **(A)** Length variation of PCGs; **(B)** GC content of the PCGs; **(C)** AT skew; **(D)** GC skew.

Among the 15 core PCGs we tested, 9 core PCGs varied in length among the eight *Amanita* species (Figure 5). The largest length variation was observed in the *rps3* gene; no two *Amanita* species contained the same length of *rps3* gene. As for the GC content, *atp9* had the highest GC content and *atp8* had the lowest content among the 15 core PCGs detected. The GC content of core PCGs varied among the different species, which indicated that the core PCGs underwent frequent base variations. According to the second parity rule, each base in the complementary DNA chain exists at a roughly equal frequency if there are no variations or selection biases (Chen H. et al., 2014). In the present study, most AT skews of core PCGs for energy metabolism were negative, except for that of the *atp8* genes from *A. brunnescens* and *A. muscaria*. The AT skew of *rps3* gene used for transcriptional regulation was positive. The GC skew of the core PCGs was variable: *atp8* gene contained a negative GC skew; *atp9* gene contained a positive GC skew; and *atp6* gene was positive in *A. basii*, *A. muscaria*, and *A. thiersii*, but negative in *A. bisporigera*, *A. pseudoporphyria*, and *A. inopinata*.

**FIGURE 6:**
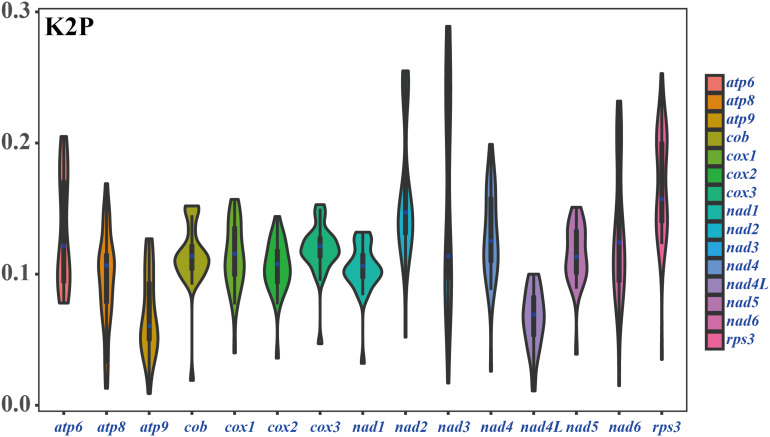
K2P genetic distance of 15 PCGs conserved in the eight *Amanita* mitogenomes.

Among the 15 core PCGs detected, the average K2P genetic distance of *atp9* and *nad4L* was the smallest, indicating that the two genes were highly conserved in *Amanita* species (Figure 6). The K2P genetic distances of *nad2*, *nad3*, and *rps3* were the highest, indicating that these genes had larger variation between different species. In addition, the K2P genetic distance of *nad3* gene varied significantly among different pairs of species, indicating the rich diversity of the *nad3* gene.

### Intron Dynamics of *cox1* Gene in *Agaricales*

We detected a total of 63 introns in the eight *Amanita* species, which belonged to two groups: group I and group II. These introns were distributed in *cox1*, *cox2*, *nad1*, *nad2*, *nadd5*, *cob*, *rns*, and *rnl* genes of mitogenomes. The number of introns in each species ranged from 0 to 22. *A. thiersii* contained the largest number of introns, whereas *A. basii* did not contain any introns. The Pearson correlation coefficient between intron sequences and mitogenome sizes was 0.97, with a *p*-value of 1 × 10^–5^, indicating that the variations of introns promoted dynamic changes in the size of *Amanita* mitogenomes. The *cox1* gene contained the most introns in *Amanita*, and 39.68% of introns were harbored in the *cox1* gene. Only one intron from the *cox1* gene belonged to the group II.

**FIGURE 7:**
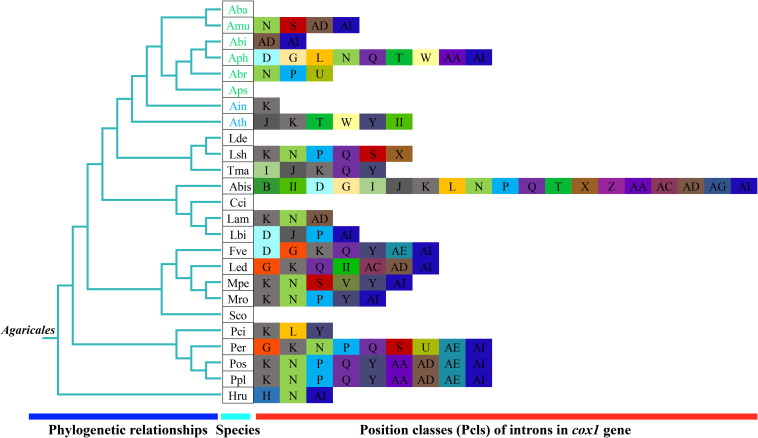
Position class (Pcl) information of *cox1* gene of the 25 *Agaricales* species. The same Pcl (orthologous intron) is represented by the same letter. The phylogenetic positions of 25 *Agaricales* species were established using the BI method and ML method based on 15 concatenated mitochondrial core proteins and two rRNA genes. The II in the figure shows the intron belongs to the group II intron. Pcls were named according to the method described by Zhang and Zhang (2019). Species IDs are given in Supplementary Table S6.

The introns in the *cox1* gene could be classified into different Pcls according to their insertion positions in the protein-coding region (Ferandon et al., 2010). We considered introns belonging to the same Pcls to be orthologous introns, with high sequence similarities. We considered introns from different Pcls to be non-homologous and they contained low sequence similarities. In the present study, we divided 25 introns in the *cox1* gene of *Amanita* into 16 Pcls (Figure 7). Among these Pcls, P615 and P1305 were the most common in *Amanita*, which were distributed in 3 of 8 *Agaricales* species. Pcls P386, P807, P867, and P1107 were distributed in 2 of the 8 *Amanita* species. The other Pcls were rare introns in *Amanita* species, which were distributed in only 1 of the 8 *Amanita* species. We detected Pcls P709 and P725 in distant species *Lyophyllum shimeji* (Li et al., 2019a), detected Pcl P206 in *Laccaria bicolor* (Li et al., 2020), and detected Pcl P234 in distant species *Agaricus bisporus* (Ferandon et al., 2010). The number and classes of Pcls from different *Amanita* species varied, which indicated that frequent intron loss/gain events occurred in the evolution of *Amanita* species. The two asymbiotic *Amanita* species contained Pcl P380, which the ectomycorrhizal *Amanita* species did not possess, suggesting that ectomycorrhizal *Amanita* species may have lost this intron classes during evolution.

## Discussion

### Size Variation of *Amanita* Mitogenomes

Until now, the mitogenome of *A. thiersii* was the largest among all the published *Agaricales* mitogenomes, and the mitogenome of *A. basii* was the smallest among the sequenced mitogenomes in *Agaricales*. The largest mitogenome in *Amanita* was 3.68 times that of the smallest mitogenome, which indicated the great variation of the *Amanita* mitogenome. These results extended our understanding of the mitogenome in *Amanita* and in *Agaricales* (Ferandon et al., 2010; Stajich et al., 2010). We identified dynamic changes in the introns as the primary factors contributing to size variations in *Amanita*. These results were consistent with previous studies (Kanzi et al., 2016; Li et al., 2019a). In addition to the quantitative variations of introns, we found that intron classes also underwent great variations in *Amanita*, which promoted additional variations in the organization and sizes among *Amanita* mitogenomes. Comparative mitogenome analysis revealed that the frequent gain/loss, as well as the possible horizontal transfer of introns, occurred in the mitogenomes of *Amanita* we detected, which enabled *Amanita* to become one of the most varied fungal group in *Agaricales*.

### Genomic Structural Variation of Ectomycorrhizal and Asymbiotic *Amanita* Species

In this study, we compared the features of mitogenomes from two saprophytic and six ectomycorrhizal *Amanita* species. We found that there were no obvious differences in genome size, GC content, tRNA, and rRNA between saprophytic and ectomycorrhizal *Amanita* species. We synthetically analyzed the repeats obtained by three methods and found that the ectomycorrhizal *Amanita* species had a high proportion of repeat sequences relative to saprophytic species. Previous studies also have found that ectomycorrhizal *Amanita* species had increased TE activities (Hess et al., 2014). The results showed that ectomycorrhizal *Amanita* species tended to have abundant repetitive elements in both nuclear genome and mitogenome, which may be related to ectomycorrhizal lifestyle adaptation. In addition, we found that ectomycorrhizal *Amanita* species had a relatively low proportion of intergenic regions, which made their mitogenomes more compact than saprophytic mitogenomes.

### Variation and Genetic Distance of Core Genes in *Amanita* Species

Most of the mitochondrial genes in eukaryotes were transferred to the nuclear genome during evolution (Adams and Palmer, 2003). Species from the Basidiomycota phylum, however, have retained the 14 core PCGs for energy metabolism and 1 *rps3* gene for transcriptional regulation (Bullerwell et al., 2000; Costa et al., 2012). In the present study, we found that all eight *Amanita* species contained the 15 core PCGs, and their length and base composition varied between different *Amanita* species. In addition, the K2P genetic distances of different PCGs also varied, which indicated the different evolution rates of core PCGs in *Amanita*. In addition to these core PCGs, some PCGs with unknown function were found in the eight *Amanita* mitogenomes. In general, the functions of *Amanita* mitogenomes were diverse and fascinating. Further studies are needed to reveal and identify the role of *Amanita* mitogenomes in growth, development, environmental adaptation, and stress resistance.

### Mitochondrial Gene Rearrangements in *Amanita* Species

Large-scale gene rearrangements were detected in the eight *Amanita* mitogenomes we tested, involving gene displacement and gene inversion. This was the first time that large-scale gene rearrangements were detected in *Amanita* species. Previous studies have shown that the accumulation of repeat sequences in fungal mitogenome promoted the mitochondrial gene rearrangement in fungi (Aguileta et al., 2014). In the present study, however, we found that *A. thiersii* and *A. inopinata*, which experienced large-scale mitochondrial rearrangements, contained fewer repetitive sequences than the other mitogenomes. Therefore, the mechanism of mitochondrial gene rearrangement in fungi needs to be further examined (Lavrov et al., 2002). *A. bisporigera* and its sister species *A. phalloides* showed significant differences in gene arrangement, indicating the variability and complexity of *Amanita* gene arrangements. The mitochondrial gene order of *A. thiersii* and *A. inopinata*, two asymbiotic *Amanita* species, varied greatly from other *Amanita* species, which indicated that they differentiated from other *Amanita* species in the early stages. The arrangement of mitochondrial genes in *Amanita* provided useful information for revealing the evolution of *Amanita* species.

### Reconstruction of Phylogenetic Relationships of Basidiomycete Species

Mitochondrial genes have become a powerful tool for the study of phylogeny, taxonomy, and population genetics of animals because of their distinct advantages (Johri et al., 2019; Li et al., 2015). Thus far, less than 100 *Basidiomycete* mitogenomes have been published, which is far less than the number of nuclear genomes available. In the present study, we first reported the eight *Amanita* mitogenomes based on the next-generation sequencing reads. On the basis of the combined mitochondrial gene set, we reconstructed the phylogenetic relationships of 66 *Basidiomycetes* and obtained tree topologies with a high support rate, which indicated that mitochondrial genes were a powerful tool for analyzing the phylogenetic relationships of fungi. From the evolutionary tree, we found that the ectomycorrhizal *Amanita* species were of single origin, and one monophyletic clade encompassed all ectomycorrhizal *Amanita* species. However, saprophytic *Amanita* species formed two distinct clades, which was not consistent with previous studies (Wolfe et al., 2012b). This phylogenetic difference may be caused by different genetic characteristics of mitochondrial and nuclear genomes (Saarma et al., 2009). More *Basidiomycete* mitogenomes need to be acquired to reveal the origin, evolution, and phylogenetic relationships of *Basidiomycete* species or other fungi.

## Data Availability Statement

The datasets generated for this study can be found in the complete mitogenomes of *A. basii*, *A. muscaria*, *A. bisporigera*, *A. phalloides*, *A. brunnescens*, *A. pseudoporphyria*, *A. inopinata*, and *A. thiersii* were deposited in the GenBank database under the accession numbers MK993555, MK993559, MK993556, MK993560, MK993557, MK993554, MK993558, and MK993561, respectively.

## Author Contributions

QL, YR, and WH conceived and designed the experiments. QL, XH, CX, and XJ performed the experiments. QL, XJ, and LP analyzed the data. QL, LP, and WH contributed reagents, materials, and analysis tools. QL wrote the manuscript. All authors contributed to the article and approved the submitted version. QL and XH revised the manuscript.

## Conflict of Interest

The authors declare that the research was conducted in the absence of any commercial or financial relationships that could be construed as a potential conflict of interest.

## Footnotes

1 https://www.ncbi.nlm.nih.gov/genome/browse#!/organelles/

2 https://www.ncbi.nlm.nih.gov/genome/browse#!/eukaryotes/

3 https://www.ncbi.nlm.nih.gov/orffinder

4 http://www.dnastar.com/
